# An Outside Description of Hay Asthma

**Published:** 1880-01

**Authors:** 


An Outside Description of Hay Asthma.—The New York *World* tells it on this wise:

Maudie Muller on an August day
Took the Fever called the Hay.
Sneezing she went, and her shrill Ah-chee!
The mock-bird echoed from the tree.
The Jedge rode slowly down the lane,
Smoothing his chestnut horse's mane,
And drew his bridle in the shade
With a sternutation to greet the maid.
He spoke of the grass, and flowers and trees,
The pollen from which makes sufferers sneeze,
And Maudie forgot her swollen nose
And even her graceful bare, brown toes,
And listened, while a pleased surprise
Looked from her watering hazel eyes.
At last, with a wild Ah-chee! Ah-chay!
Ah-choo! Achaw! he rode away.
Maude Muller looked and sneezed, "Ah-chee!
That I the Jedge's bride might be!
He would dress me with silks and diamond rings,
And take me up to the White Mountings.

And I'd use the finest cambric *mouchoir*,
And never have the Hay fever more."
The Jedge looked back as he climbed the hill,
And heard her sternutations shrill.
" Would she were mine, and I to-day
Were rid of this dab Fever of the Hay!"
But closing his heart, the Jedge rode on,
And Maudie was left in the field alone.
Then she took up her burden of life anew,
Sighing only, " Ah-chee! Ah-choo!
Of all sad words of tongue or pen,
The saddest are "Hay-fever time again!"
Ah! well for us that a region lies
Where the infusoria never rise;
And in the hereafter angels may
Find a cure for the Fever called the Hay!

